# The reduced PDCD5 protein is correlated with the degree of tumor differentiation in endometrioid endometrial carcinoma

**DOI:** 10.1186/s40064-016-2698-z

**Published:** 2016-07-07

**Authors:** Meng Gao, Wei Gao, Zhanying Wang, Yanping Liu, Yue Li, Chao Wei, Yingshuo Sun, Chun Guo, Lining Zhang, Zengtao Wei, Xiaoyan Wang

**Affiliations:** Department of Immunology, Shandong University School of Medicine, 44# Wenhua Xi Road, Jinan, 250012 Shandong People’s Republic of China; Department of Clinical Laboratory Services, Linyi People’s Hospital, Linyi, Shandong People’s Republic of China; Department of Gynecology, Jinan Central Hospital Affiliated to Shandong University, Jinan, Shandong People’s Republic of China; Department of Gynecology and Obstetrics, Shandong University School of Medicine, 44# Wenhua Xi Road, Jinan, 250012 Shandong People’s Republic of China; Department of Pathology, The Fourth Hospital of Jinan City, Jinan, Shandong People’s Republic of China

**Keywords:** PDCD5, Expression, Endometrial cancer, Differentiation

## Abstract

Endometrial cancer is one of the most common malignancies in the female genital tract. Programmed cell death 5 (PDCD5) is a newly identified apoptosis related gene and plays an important role in the development of some human tumors. However, the expression and clinical significance of PDCD5 in endometrial cancer have not been fully elucidated. Here, we evaluated the expression of PDCD5 in endometrioid endometrial carcinoma and control endometrium by qRT-PCR, western blot and immunohistochemistry, and analyzed the associations of PDCD5 expression with clinicopathological parameters of patients. In addition, we detected the expression of PDCD5 in control endometrial glandular epithelial cells and endometrioid endometrial carcinoma-derived cell line KLE by immunocytochemistry. The results showed that PDCD5 protein mainly expressed in the cytoplasm of glandular epithelial cells and endometrial carcinoma cells, and there was a low level of PDCD5 expression in the nuclei of the above cells. Furthermore, PDCD5 protein level was significantly lower in endometrial carcinoma samples than that in control endometrium. The decreased PDCD5 expression was correlated with the tumor differentiation degree. It is clear that PDCD5 protein expression was lower in middle and low differentiated endometrial carcinoma compared with control endometrium and high differentiated endometrial carcinoma. However, there were no significant differences of PDCD5 expression between the proliferative phase and the secretory phase of control endometrium, as well as between high differentiated endometrial carcinoma and controls. The results were verified in control glandular epithelial cells and KLE cells by immunocytochemistry. Therefore, PDCD5 may play a key role in the pathogenesis of endometrial cancer and may be a novel target for diagnosis and treatment of endometrial cancer.

## Background

Programmed cell death 5 (PDCD5) is a novel apoptosis accelerating gene which was first identified from the leukaemic cell clone TF-1 cells undergoing apoptosis in Human Disease Gene Center of Peking University. Therefore, it is also called TF-1 cell apoptosis-related gene 19 (TFAR19) (Liu et al. 1999). It has been reported that PDCD5 promotes DNA damage-induced apoptosis by interacting with Tip60 (a histone acetyltransferase) (Xu et al. 2009), and PDCD5 phosphorylation induced by a multifunctional kinase CK2 is an important process for its apoptotic potential (Salvi et al. 2009). The functions of PDCD5 are also related to tumor suppressor gene P53. Park et al. reported YY1-associated factor 2 (YAF2) interacts with PDCD5 and promotes TP53-mediated genotoxic stress response via stabilization of PDCD5. OTU deubiquitinase (OTUD5) binds to PDCD5 in response to etoposide treatment and effectively mediates the sequential activation of both PDCD5 and P53 (Park et al. 2015a, b). Xu et al. found that PDCD5 interacts with p53 and functions as a positive regulator in the p53 pathway (Xu et al. 2012b). Choi et al. demonstrated that PDCD5 selectively mediates HDAC3 dissociation from p53, which induces HDAC3 cleavage and ubiquitin-dependent proteasomal degradation (Choi et al. 2015). Cui et al. suggested that the expression of PDCD5 is negatively regulated by DNAJB1 and DNAJB1 targets PDCD5 to suppress p53-dependent apoptosis of cancer cells (Cui et al. 2015). The studies have confirmed that PDCD5 also suppresses tumorigenesis by inhibiting the Ras/Raf/MEK/ERK signaling pathway in the human osteosarcoma cell line MG-63 (Han et al. 2012) and adenovirus carrying PDCD5 gene exerts potent antitumor efficacy on common human leukemic cell lines (Xie et al. 2009). In addition, the downregulation of PDCD5 has been observed in multiple types of cancers, such as breast cancer (Hedenfalk et al. 2001), ovarian carcinoma (Zhang et al. 2011), gastric cancer (Yang et al. 2006), hepatocellular carcinoma (Fu et al. 2013; Xu et al. 2001), acute and chronic myeloid leukemia (Ruan et al. 2006), glioma (Li et al. 2008) and laryngeal squamous cell carcinoma (Xu et al. 2013), and correlates with tumor progression and prognosis. PDCD5 can also enhances chemosensitivity of tumor cells (Wang et al. 2013; Xu et al. 2012a), and negatively regulates inflammation in autoimmune diseases (Xiao et al. 2013, 2015a, b). These findings suggest that apoptosis-related gene PDCD5 could be acted as tumor suppressor gene and inflammation-relative gene, and play important roles in the pathogenesis of cancers and inflammatory diseases.

Endometrial cancer is one of the most common malignancies in female reproductive system (Horn et al. 2007; Wang et al. 2012). Epidemiologic studies show that some risk factors, including advanced age, early menarche, late menopause, nulliparity, long-term use of estrogen, obesity and diabetes, are associated with an increased risk of endometrial cancer (Purdie and Green 2001). The vast majority of endometrial cancers are carcinomas which originate from the single layer of epithelial cells. Endometrial carcinomas are broadly organized into two categories, Type I and Type II, based on clinical features and pathogenesis. Type I endometrial carcinomas occur most commonly before and around the time of menopause, and represent 75–90 % of endometrial cancer. They are often endometrioid carcinomas, estrogen-dependent, and have a favorable treatment outcome. Type II endometrial carcinomas usually occur in older, post-menopausal people. They are often non-endometrioid carcinomas (such as serous carcinoma, clear cell carcinoma and mucinous carcinoma etc.), estrogen-independent and carry a poorer prognosis (Sherman 2000; Bokhman 1983; Hecht and Mutter 2006; Emons et al. 2000). Nowadays, numerous tumor suppressor genes and oncogenes, such as PTEN and K-ras have been reported to be involved in the development of endometrial cancer (Arafa et al. 2010; Ellis and Ghaem-Maghami. 2010; Honkavuori-Toivola et al. 2012; Angioli et al. 2013). However, the expression of PDCD5 and its clinical significance in endometrial cancer have not been fully elucidated.

In the present study, we investigated the levels of PDCD5 expression by qRT-PCR, western blot, immunohistochemistry and immunocytochemistry in endometrioid endometrial carcinoma and control endometrium tissues, as well as control endometrial glandular epithelial cells and endometrial cancer cell line KLE. Furthermore, we analyzed the associations of PDCD5 expression with clinical and pathological characteristics of patients with endometrioid endometrial carcinoma. The results showed that PDCD5 protein expression was decreased in endometrioid endometrial carcinoma tissues and correlated with the degree of tumor differentiation, suggesting that PDCD5 could participate in the development and progression of endometrioid endometrial carcinoma.

## Results

### The expression levels of PDCD5 mRNA in control endometrium and endometrioid endometrial carcinoma tissues detected by qRT-PCR

**Fig. 1:**
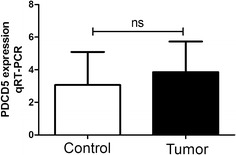
The expression of PDCD5 mRNA in endometrioid endometrial carcinoma and control endometrium detected by qRT-PCR. The levels of PDCD5 mRNA in freshly frozen endometrioid endometrial carcinoma and control endometrium were detected by qRT-PCR. Data were normalized to GAPDH. There were no significant differences between the two groups (P > 0.05)

In order to study the roles of PDCD5 in endometrioid endometrial carcinoma, we firstly detected PDCD5 mRNA expression in 16 freshly frozen endometrioid endometrial carcinoma tissues and 17 control endometrium using qRT-PCR. The results showed that there were no significant differences in PDCD5 mRNA expression between endometrioid endometrial carcinoma tissues and control endometrium (P > 0.05) (Fig. 1).

### The expression levels of PDCD5 protein in control endometrium and endometrioid endometrial carcinoma tissues detected by western blot

**Fig. 2:**
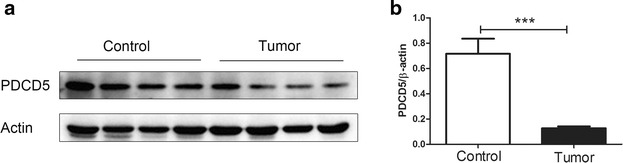
The expression of PDCD5 protein in endometrioid endometrial carcinoma and control endometrium detected by western blot. **a** The levels of PDCD5 protein in freshly frozen endometrioid endometrial carcinoma and control endometrium were detected by western blot. β-actin was used as control. **b** The expression of PDCD5 protein was significantly lower in endometrioid endometrial carcinoma tissues than that in control endometrium (P < 0.001). ***P < 0.001

To further explore the expression status of PDCD5 in endometrioid endometrial carcinoma, we detected the levels of PDCD5 protein in 16 freshly frozen endometrioid endometrial carcinoma tissues and 17 control endometrium using western blot. The data showed that the expression of PDCD5 protein in endometrioid endometrial carcinoma tissues was significantly lower than that in control endometrium, which was inconsistent with the results of qRT-PCR (P < 0.001) (Fig. 2a, b).

### The expression levels of PDCD5 protein in control endometrium and endometrioid endometrial carcinoma tissues detected by IHC

**Fig. 3:**
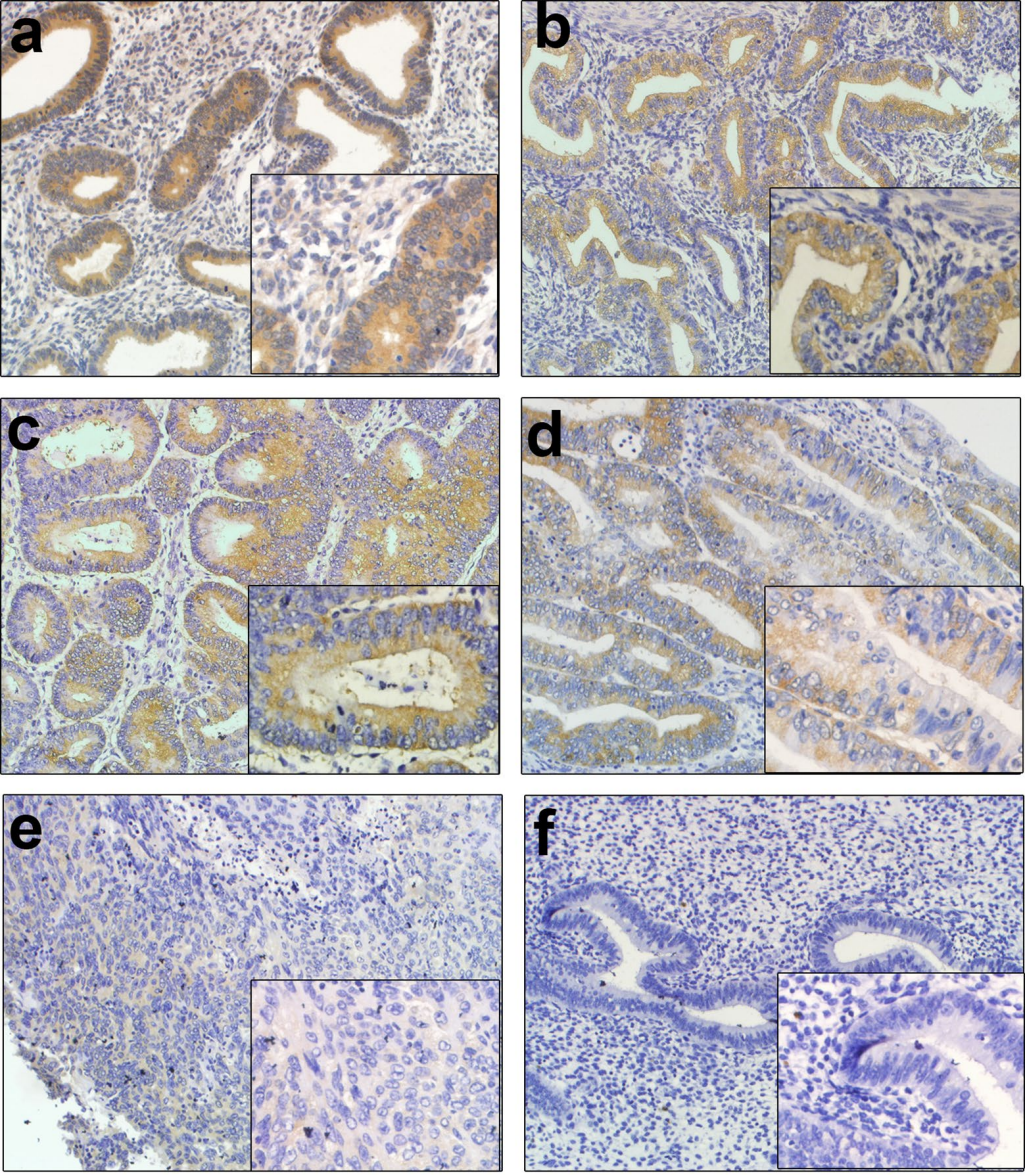
Immunohistochemical staining for PDCD5 protein in endometrioid endometrial carcinoma and control endometrium (magnification, ×200, ×400). **a** The immunostaining of PDCD5 protein in the proliferative phase of control endometrium; **b** the immunostaining of PDCD5 protein in the secretory phase of control endometrium; **c** the immunostaining of PDCD5 protein in high differentiation of endometrioid endometrial carcinoma tissues; **d** the immunostaining of PDCD5 protein in middle differentiation of endometrioid endometrial carcinoma tissues; **e** the immunostaining of PDCD5 protein in low differentiation of endometrioid endometrial carcinoma tissues; **f** negative control

**Fig. 4:**
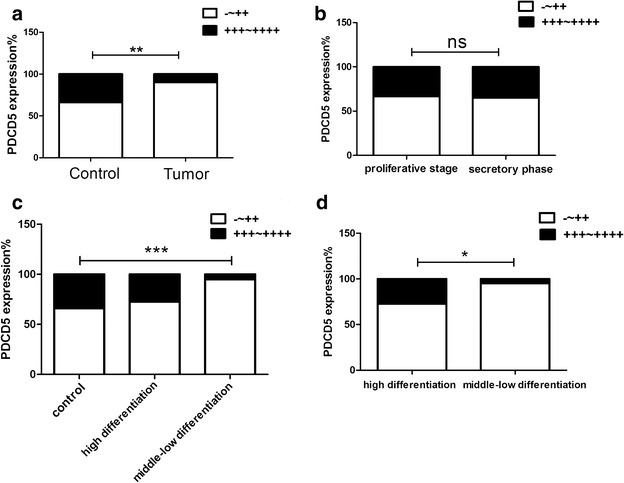
Statistical analysis of PDCD5 expression in 51 endometrioid endometrial carcinoma specimens and 53 control endometrium. **a** The staining index of PDCD5 in endometrial carcinoma specimens was significantly lower than that in control endometrium (P < 0.01); **b** No significant differences in PDCD5 expression were observed between proliferative phase and secretory phase of control endometrium (P > 0.05); **c** The staining index of PDCD5 in middle-low differentiation of endometrial carcinoma was significantly lower than that in control endometrium (P < 0.001), but there were no obvious differences between high differentiation of endometrial carcinoma and control endometrium; **d** The staining index of PDCD5 in high differentiation of endometrial carcinoma was significantly higher than that in middle-low differentiation of endometrial carcinoma (P < 0.05). *P < 0.05; **P < 0.01; ***P < 0.001

To verify the results of western blot, we next determine the levels of PDCD5 protein expression in 51 endometrioid endometrial carcinoma tissues and 53 control endometrium using IHC staining. In accordance with the results of western blot, the levels of PDCD5 protein were significantly decreased in endometrioid endometrial carcinoma tissues compared with control endometrium (P < 0.01) (Figs. 3a–e, 4a), which suggested that the levels of PDCD5 mRNA and protein are inconsistent in endometrioid endometrial carcinoma tissues.

The endometrial lining undergoes cyclic regeneration and displays menstrual cycle (proliferative, secretory and menstrual phase) under the influence of estrogen and progesterone. Among 53 cases of control endometrium, 30 cases were proliferate phase, 23 cases were secretory phase. However, our results showed that there were no significant differences in PDCD5 expression between proliferative phase and secretory phase of the control endometrium (P > 0.05) (Figs. 3a, b, 4b).

In addition, endometrioid endometrial carcinoma is divided into three types, such as high differentiation, middle differentiation and low differentiation according to their differentiation degree. Therefore, we further analyzed the differences in PDCD5 protein expression between control endometium and high differentiation or middle-low differentiation of endometrioid endometrial carcinoma samples. The results showed that PDCD5 protein expression was significantly decreased in middle-low differentiation of endometrioid endometrial carcinoma tissues compared with control endometrium (P < 0.001), but there was no obvious difference between high differentiation of endometrioid endometrial carcinoma tissues and control endometrium (Figs. 3a–e, 4c).

### The expression sites of PDCD5 protein in control endometrium and endometrioid endometrial carcinoma tissues

**Fig. 5:**
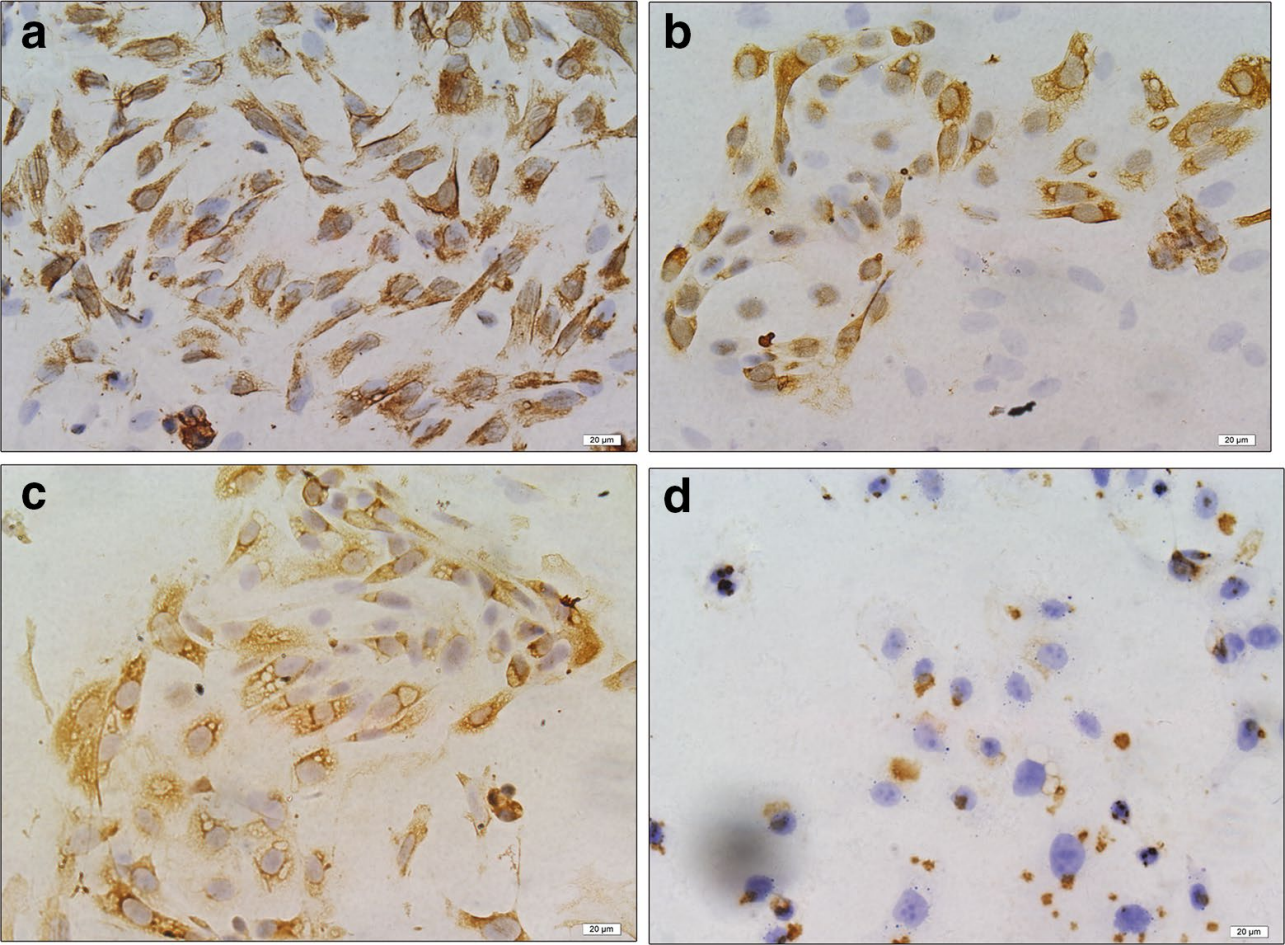
Immunocytochemical staining for vimentin, cytokeratin and PDCD5 protein in endometrial stromal cells, glandular epithelial cells and KLE cells (magnification, ×400). **a** The immunostaining of vimentin in endometrial stromal cells; **b** the immunostaining of cytokeratin in endometrial glandular epithelial cells; **c** the immunostaining of PDCD5 protein in endometrial glandular epithelial cells; **d** the immunostaining of PDCD5 protein in KLE cells

The results from IHC revealed PDCD5 protein was mainly located in the cytoplasm of the control endometrial glandular cells or endometrioid endometrial carcinoma cells, and there was also a low level of PDCD5 expression in the nuclei of these cells (Fig. 3a–e). In order to further confirm the results, we isolated and identified control endometrial glandular cells and stromal cells, and then detected the PDCD5 expression in control endometrial glandular epithelial cells and stromal cells, as well as endometrioid endometrial carcinoma cell line KLE. The results showed that stromal cells expressed vimentin and glandular epithelial cells expressed cytokeratin (Figs. 5a, b). More importantly, we found that PDCD5 positive staining was indeed mainly located in the cytoplasm of control endometrial glandular epithelial cells and KLE cells, and weak PDCD5 expression was also found in the nuclei of the above cells. However, no obvious positive staining could be observed in stromal cells. Furthermore, PDCD5 protein expression in KLE cells was weaker than that in control endometrial glandular epithelial cells (Fig. 5c, d).

### The associations of PDCD5 protein expression in endometrioid endometrial carcinoma tissues with clinicopathologic parameters of patients

In order to explore the clinical significance of PDCD5 expression in endometrioid endometrial carcinoma, we analyzed the relationships between PDCD5 expression levels and the clinicopathological parameters. As shown in Table 1, there were no significant correlations between PDCD5 protein levels and age, myometrial invasion, FIGO stage, estrogen receptor or progestin receptor. However, we found that the expression of PDCD5 was significantly associated with the degree of tumor differentiation (P < 0.05). PDCD5 protein levels were decreased in middle-low differentiation of endometrioid endometrial carcinoma samples compared with high differentiation of endometrioid endometrial carcinoma samples (P < 0.05) (Fig. 4d), which suggested that PDCD5 expression might be correlated with the progression of endometrioid endometrial carcinoma.

**Table 1:** Association analysis of PDCD5 expression in endometrioid adenocarcinoma tissues and clinicopathologic parameters

Clinical and pathological features	PDCD5 expression			
	n	Negative-moderate	Strong	P value
Age				
≤55	20	17	3	0.3359
>55	30	28	2	
Myometrial invasion (%)				
≤50	31	27	4	0.8192
>50	19	18	1	
FIGO stage				
IA–IB	33	29	4	0.4861
IC–II	17	16	1	
Tumor differentiation degree				
High differentiation	11	8	3	0.0278*
Middle-low differentiation	40	38	2	
Estrogen receptor				
Negative-weak	11	10	1	0.7703
Moderate-strong	31	29	2	
Progestin receptor				
Negative-weak	11	10	1	0.7703
Moderate-strong	31	29	2	

* P<0.05

## Discussion

PDCD5 was initially identified as a novel apoptosis-related gene (Tracy et al. 2014). Recent studies showed that PDCD5 might be a new tumor suppressor gene and involved in tumor development and progression (Xu et al. 2012a). It has been reported that PDCD5 mRNA and protein were both downregulated in some human tumor tissues such as breast cancer (Hedenfalk et al. 2001), ovarian carcinoma (Gao et al. 2015; Zhang et al. 2011), gastric cancer (Yang et al. 2006), hepatocellular carcinoma (Xu et al. 2001), acute and chronic myeloid leukemia (Ruan et al. 2006), glioma (Li et al. 2008) and laryngeal squamous cell carcinoma (Xu et al. 2013). However, it remains not very clear about PDCD5 expression in endometrial cancer. In the present study, we demonstrated that PDCD5 protein expression was decreased in endometrioid endometrial carcinoma tissues compared with control endometrium by western blot. However, unlike other tumors, PDCD5 mRNA levels had no apparent change between endometrioid endometrial carcinoma tissues and control endometrium.

To date, the regulation mechanisms of abnormal PDCD5 expression have not been fully clarified. Fernandez et al. reported that bisphenol A exposure induced the downregulation of PDCD5 expression in human breast epithelial cells after accompanied by DNA methylation (Fernandez et al. 2012). These data suggested that the abnormal expression of PDCD5 might be associated with DNA methylation. We speculate that the differences in PDCD5 mRNA and protein levels may be because the expression of PDCD5 could have post-transcriptional regulation in endometrioid endometrial carcinoma. However, the exact regulatory mechanism needs further exploration.

The endometrium is affected by ovarian hormones and undergoes proliferative, secretory and menstrual phase. These ovarian hormones including estrogen and progesterone could regulate the expression of some genes by hormone receptors. Ma et al. reported that the expression of HOXA-10 was regulated by estrogen and progesterone in adult uterus. Progesterone could promote the expression of HOXA-10, but estrogen could inhibit its expression (Ma et al. 1998). This explained the difference in HOXA-10 expression between proliferative phase and secretory phase of endometrium. To explore whether PDCD5 expression is also regulated by ovarian hormones, we compared the expression of PDCD5 in proliferative phase and secretory phase of control endometrium. However, there were no significant differences between them. These results suggest that PDCD5 expression may not be regulated by ovarian hormones.

PDCD5 is a novel protein related to regulation of cell apoptosis, its expression level is significantly increased in cells undergoing apoptosis, and then the protein translocates rapidly from the cytoplasm to the nuclei of cells (Chen et al. 2001; Li et al. 2016). The nuclear translocation of PDCD5 is a universal earlier event of the apoptotic process, and may be a novel early marker for apoptosis.

To explore the subcellular localization of PDCD5 in control endometrium and endometrioid endometrial carcinoma tissues, we detected PDCD5 expression by immunohistochemistry analysis and found that PDCD5 staining mainly existed in the cytoplasm of the control endometrial glandular cells or endometrioid endometrial carcinoma cells, and weak PDCD5 expression was also observed in the nuclei of these cells. Immunocytochemistry analysis of control endometrial glandular cells and endometrioid endometrial carcinoma cell line KLE further confirmed these results. This is consistent with the report of Li et al., they found that PDCD5 was predominantly expressed in the cytoplasm of glioma cells, and a low level of PDCD5 was also detected in the nuclei (Li et al. 2008).

Previous reports showed that the decreased expression of PDCD5 significantly correlated with the high-grade tumor in renal clear cell carcinomas (Tan et al. 2006) and gliomas (Li et al. 2008). Moreover, the reduced expression of PDCD5 was related to short survival periods of patients with gastric cancer (Yang et al. 2006) and ovarian serous carcinoma (Zhang et al. 2011). These results suggest that PDCD5 is not only an apoptosis-related gene, but also a tumor suppressor.

In this study, we found that PDCD5 protein levels were not related to age, myometrial invasion, FIGO stage, estrogen receptor and progestin receptor of patients with endometrioid endometrial carcinoma. However, the expression of PDCD5 was significantly associated with the degree of tumor differentiation. The levels of PDCD5 protein were downregulated in middle-low differentiation of endometrioid endometrial carcinoma samples compared with high differentiation endometrioid endometrial carcinoma samples. This may be the reason that PDCD5 protein was significantly lower in middle-low differentiation of endometrioid endometrial carcinoma tissues than that in control endometrium, but there was no significant difference in PDCD5 expression between high differentiation of endometrioid endometrial carcinoma tissues and control endometrium. Zhang et al. reported that lost or decreased PDCD5 expression was associated significantly with FIGO stage in ovarian serous carcinomas (Zhang et al. 2011) and Gao et al. reported that the decreased expression of PDCD5 proteins was significantly associated with the tumor size and mitosis of gastrointestinal stromal tumors (Gao et al. 2012). The discrepancy may be due to clinicopathological characteristics of different subjects.

## Conclusion

In the current study, we found that PDCD5 protein was reduced in endometrioid endometrial carcinoma tissues and its levels were associated with the degree of tumor differentiation. The results suggested that PDCD5 expression might have an important role in the development and progression of endometrioid endometrial carcinoma and might contribute to the improvement of prognosis. However, the detailed regulation mechanism of decreased PDCD5 expression in endometrioid endometrial carcinoma requires further investigation.

## Methods

### Sample collection

Sixteen freshly frozen endometrioid endometrial carcinoma tissues and 51 paraffin-embedded endometrioid endometrial carcinoma specimens were obtained from patients aged 44–74 years who underwent primary surgeries at Department of Gynecology and Obstetrics, Jinan Central Hospital affiliated to Shandong University from 2012 to 2014. The clinical stage was assessed according to the International Federation of Gynecology and Obstetrics (FIGO) system (2009). Tumor differentiation degree, depth of myometrial invasion (MI), the expression of estrogen receptor (ER) and progestrone receptor (PR) were also evaluated. The basic characteristics of endometrioid endometrial carcinoma patients are listed in Table 1. Seventeen freshly frozen control endometrium tissues and 53 paraffin-embedded control endometrial specimens were also collected from patients aged 40–78 years who underwent uterine curettage for other diseases other than endometrial cancer. According to their menstrual history and histopathological examination, the control group was categorized as proliferative and secretory phases. This study and the collection of all human samples were approved by the Institutional Ethics Committee of Shandong University and all of the patients gave their informed consent.

### RNA isolation and quantitative real-time PCR (qRT-PCR)

Total RNA was extracted from frozen tissues using a modified TRIzol (TIANGEN, Beijing, China) one-step extraction method. cDNA was synthesized using the FastQuant RT Kit (TIANGEN, Beijing, China) according to the manufacturer’s instructions. Quantitative real-time PCR was performed in 20 µL volume containing 10 µL of UltraSYBR Mixture (CWBIO, Beijing, China), 0.2 µL of cDNA, and 1 µL of primer (PDCD5 and GADPH). The sequences of the primers are as follows: PDCD5: sense, 5′-GTT CTG GAT CAG TCG GCC C-3′, and antisense, 5′-TCG TCA TCT TCA TCA GAG TCC A-3′; GAPDH: sense, 5′-AAC GGA TTT GGT CGT ATT GGG-3′, and antisense, 5′-CCT GGA AGA TGG TGA TGG GAT-3′. Polymerase chain reaction was performed according to the following programs: denatured at 95 °C for 10 min, and then followed by 39 cycles of 95 °C for 15 s, 60 °C for 1 min and 65 °C for 5 s to stop the reaction. Each sample was conducted in triplicate. The results were analyzed using the 2 − ΔΔCt method.

### Western blot analysis

The proteins were extracted from freshly frozen endometrioid endometrial carcinoma and control endometrium tissues using Radio Immunoprecipitation Assay (RIPA) buffer containing 1 % Phenylmethanesulfonyl fluoride (PMSF) and 0.5 % phosphatase inhibitor (PI). Then the bicinchoninic acid protein assay kit (Thermo Scientific, Rockford, IL, USA) was used to measure the protein concentrations. Each sample (40 µg) was analyzed by 15 % sodium dodecyl sulfate–polyacrylamide gel and then transferred onto a PVDF membrane. Then the membrane was blocked by 5 % bovine serum albumin for 2 h. The membrane was incubated overnight at 4 °C with 1:1000 dillution of PDCD5 (Abcam, Shanghai, China) and β-actin (ZSJQB Co., Ltd. Beijing, China) antibodies followed by horseradish peroxidase conjugated second antibodies (1:2000 dilution, ZSJQB Co., Ltd. Beijing, China) for 1 h at room temperature. After washing, the signals were visualized by an ECL (Amersham Biosciences, Little Chalfont, UK) western blotting detection system according to the manufacturer’s instruction. Western blot was performed at least three times for each sample.

### Immunohistochemistry (IHC)

The paraffin sections (4 µm) were dewaxed and rehydrated by infiltrating in dimethylbenzene twice and graded ethanols. After antigen microwave retrieval and endogenous peroxidase blocking, the slides were blocked by 10 % goat serum for 15 min at 37 °C. Then the sections were incubated with 1:1000 dillution of PDCD5 antibody (abcam, Shanghai, China) overnight in a wet chamber at 4 °C followed by secondary antibody conjugated with horseradish peroxidase (ZSJQB Co., Ltd. Beijing, China) for 30 min at 37 °C. Then the expression of PDCD5 was visualized using diaminobenzidine (DAB) kit (Zsbio, Beijing, China) and the nuclei were counterstained with hematoxylin. Equal volume of PBS was used instead of PDCD5 antibody and served as a negative control. Each sample was performed in duplicate.

All IHC staining was analyzed by two experienced pathologists independently. The staining intensity was scored from 0 to 3 (0, no staining; 1, weak; 2, moderate; 3, strong). Based on the percentage of positive cells, the staining extent was scored from 0 to 4 (0, <1 %; 1, 1–10 %; 2, 11–50 %; 3, 51–80 %; 4, 81–100 %). For each slide, the two scores were combined to produce a final grade of PDCD5 expression: 0 (−), 1–3 (+), 4 and 6 (++), 8 and 9 (+++), 12 (++++). The expression of PDCD5 was defined as follows: −, + and ++ were classified as low and middle expression, whereas +++ and ++++ was graded as high expression.

### The isolation of glandular epithelial cells and stromal cells derived from control endometrium

The glandular epithelial cells and stromal cells derived from secretory phase of control endometrium were isolated according to the method described previously (Sugawara et al. 1997) with slight modifications. Briefly, the endometrial tissues were suspended in a sterile tube containing DMEM/F12 1:1 (HyClone Corporation, Beijing, China), 10 % FBS (Gibco, CA, USA), 100 U/ml of penicillin and 100 U/ml of streptomycin. And then the specimens were minced and digested in 0.25 % collagenase type IA (Gibco, CA, USA) at 37 °C in a shaking water bath for about 50 min. Two to three volumes of pre-warmed culture medium was used to stop the collagenase activity. The cell suspension was successively filtered through a 154-mm monofilament cupreous mesh and a 38.5-mm monofilament cupreous mesh. The filtrate containing stromal cells was collected and centrifuged at 1000 rpm/min for 10 min, the pellet was resuspended and incubated in 24-well plates with cover slips at 2.5 × 10^5^ cells/well at 37 °C in 95 % air and 5 % CO_2_. To remove the non-attached cells, the culture medium was replaced with fresh medium 2 h later. The 38.5 mm monofilament cupreous mesh was washed thoroughly upside down with culture medium, then the cell suspension containing glandular epithelial cells was collected and centrifuged at 1000 rpm/min for 10 min. The pellet was resuspended and incubated in 24-well plates with cover slips at 5 × 10^4^ cells/well at 37 °C in 95 % air and 5 % CO_2_.

### Immunocytochemistry (ICC)

The human endometrioid endometrial carcinoma cells (KLE, low differentiation) were purchased from China Center for Type Culture Collection (Wuhan, China) and cultured in DMEM/F12 1:1 (HyClone Corporation, Beijing, China) containing 10 % fetal bovine serum (Gibco, CA, USA), 100 U/ml of penicillin and 100 U/ml of streptomycin. The cells were seeded in 24-well plates with cover slips at 5 × 10^4^ cells/well at 37 °C in 95 % air and 5 % CO_2_. After the glandular epithelial cells, stromal cells and KLE cells were cultured in 24-well plates with cover slips overnight, the cells on the cover slip were fixed with 4 % paraformaldehyde for 30 min. After successively endogenous peroxidase blocking and 10 % goat serum blocking, the cells were respectively incubated with anti-human antibodies against vimentin (1:100) (ZSJQB Co., Ltd. Beijing, China), cytokeratin (1:100) (ZSJQB Co., Ltd. Beijing, China) and PDCD5 (1:1000) (abcam, Shanghai, China) overnight at 4 °C. The cells were washed with PBS and incubated with horseradish peroxidase-conjugated secondary antibodies (ZSJQB Co., Ltd. Beijing, China) for 30 min at 37 °C. Then the protein expression was visualized using diaminobenzidine (DAB) kit (Zsbio, Beijing, China) and the nuclei were counterstained with hematoxylin. Each sample was performed in duplicate.

### Data analysis

The unpaired Student’s t test was used to analyze the expression of PDCD5 detected by qRT-PCR. The Gel-Pro analyzer was used to detect the grey value of the bands of PDCD5 expression detected by western blot, and then the unpaired Student’s t test was used for analysis. The immunohistochemical data was analyzed using chi^2^ test. And the GraphPad Prism 5 (GraphPad Software Inc., CA, USA) was used for statistical analysis. All data were expressed as mean ± SEM. P < 0.05 was considered statistically significant.

## Abbreviations

cDNA: complementary deoxyribonucleic acid
DAB: diaminobenzidine
ER: estrogen receptor
FIGO: International Federation of Gynecology and Obstetrics
ICC: immunocytochemistry
IHC: immunohistochemistry
MI: myometrial invasion
PDCD5: programmed cell death 5
PI: phosphatase inhibitor
PMSF: phenylmethanesulfonyl fluoride
PR: progestrone receptor
qRT-PCR: quantitative real-time polymerase chain reaction
TFAR19: TF-1 cell apoptosis-related gene 19
